# Synergistic Action of Genistein and Calcitriol in Immature Osteosarcoma MG-63 Cells by SGPL1 Up-Regulation

**DOI:** 10.1371/journal.pone.0169742

**Published:** 2017-01-26

**Authors:** Nadja Engel, Anna Adamus, Nicolas Schauer, Juliane Kühn, Barbara Nebe, Guido Seitz, Karin Kraft

**Affiliations:** 1 Department of Pediatric Surgery, University Hospital Marburg, Baldingerstraße, Marburg, Germany; 2 Department of Cell Biology, Rostock University Medical Center, Schillingallee, Rostock, Germany; 3 Metabolomic Discoveries GmbH, Am Mühlenberg, Potsdam-Golm, Germany; 4 Complementary Medicine, Center of Internal Medicine, Rostock University Medical Center, Ernst-Heydemann-Straße 6, Rostock, Germany; University of Crete, GREECE

## Abstract

**Background:**

Phytoestrogens such as genistein, the most prominent isoflavone from soy, show concentration-dependent anti-estrogenic or estrogenic effects. High genistein concentrations (>10 μM) also promote proliferation of bone cancer cells *in vitro*. On the other hand, the most active component of the vitamin D family, calcitriol, has been shown to be tumor protective *in vitro* and *in vivo*. The purpose of this study was to examine a putative synergism of genistein and calcitriol in two osteosarcoma cell lines MG-63 (early osteoblast), Saos-2 (mature osteoblast) and primary osteoblasts.

**Methods:**

Thus, an initial screening based on cell cycle phase alterations, estrogen (ER) and vitamin D receptor (VDR) expression, live cell metabolic monitoring, and metabolomics were performed.

**Results:**

Exposure to the combination of 100 μM genistein and 10 nM calcitriol reduced the number of proliferative cells to control levels, increased ERß and VDR expression, and reduced extracellular acidification (40%) as well as respiratory activity (70%), primarily in MG-63 cells. In order to identify the underlying cellular mechanisms in the MG-63 cell line, metabolic profiling via GC/MS technology was conducted. Combined treatment significantly influenced lipids and amino acids preferably, whereas metabolites of the energy metabolism were not altered. The comparative analysis of the log2-ratios revealed that after combined treatment only the metabolite ethanolamine was highly up-regulated. This is the result: a strong overexpression (350%) of the enzyme sphingosine-1-phosphate lyase (SGPL1), which irreversibly degrades sphingosine-1-phosphate (S1P), thereby, generating ethanolamine. S1P production and secretion is associated with an increased capability of migration and invasion of cancer cells.

**Conclusion:**

From these results can be concluded that the tumor promoting effect of high concentrations of genistein in immature osteosarcoma cells is reduced by the co-administration of calcitriol, primarily by the breakdown of S1P. It should be tested whether this anti-metastatic pathway can be stimulated by combined treatment also in metastatic xenograft mice models.

## Background

Phytoestrogens, e.g. the well investigated isoflavone genistein are able to prevent and to reduce the development of breast cancer *in vitro* and *in vivo* [1, 2]. This effect is due to structural similarities to the endogenous steroid hormone 17ß-estradiol, whereby they can trigger both estrogenic and antiestrogenic effects via binding to the estrogen receptors ERα and/or ERß [3]. This finding has led to intense discussions on the safety of phytoestrogens. *In vitro* as well as *in vivo* studies have demonstrated that genistein enhanced the proliferation of estrogen-dependent human breast cancer cells (MCF-7) already at low concentrations (10 nM), 100 nM achieved proliferative effects similar to those of 1 nM estradiol [4, 5]. However, high concentrations beyond 10 μM inhibited cell proliferation and induced apoptosis of estrogen-sensitive breast cancer cells, most likely by inhibiting the intrinsic tyrosine kinase activity of growth factor receptors [6]. Furthermore, high concentrations of genistein and other soy isoflavones stimulate growth of bone and metastatic breast cancer [7–9]. Due to these effects, isolated phytoestrogens are not recommended for dietary consumption in the case of breast and bone tumors, detected previously.

Despite recent advances in treatment of breast cancer, still substantial numbers of patients develop metastatic disease, especially in the bones up to 70% [10]. Breast cancer is the most common source of bone metastasis which is often characterized by an estrogen-positive phenotype: 65% of the lesions are lytic, 10% are blastic, and 25% consist of both lytic and blastic lesions.

The most biologically active hormonal form of vitamin D3, calcitriol (1α,25(OH)_2_Vitamin D_3_), is synthesized endogenously by a series of reactions, starting with UVB radiation on human skin, and followed by stepwise hydroxylation in liver and kidney. Potential vitamin D target tissues (e.g. colon, prostate, breast, lung, pancreas) can also synthesize and degrade calcitriol. Local production and degradation of calcitriol have been suggested to represent a key factor in several types of human cancer. The function of the vitamin D complex for human body and health is widespread, from effects on cellular differentiation and proliferation and on central nervous system up to the modulation of immune responsiveness [11]. *In vivo* the results are less convincing, often conflicting and show considerable variability [11]. However, the results of *in vivo* studies suggest that the calcitriol precursor cholecalciferol could act as a chemopreventive agent against several malignancies, as an association between low serum levels of the calcitriol precursor calcidiol (25(OH)D) and an increased incidence and mortality of several types of tumors such as non-Hodgkin’s lymphoma, melanoma, breast, prostate, colorectal, ovarian, kidney, esophagus, and stomach cancer was confirmed [12–14]. Recently, Keum and Giovannucci [15] have published that supplementation with cholecalciferol at doses of up to 800 IU per day presumably has no substantial effect on cancer incidence within 2–7 years, but is related to a statistically significant 12% reduction in cancer mortality.

Up to now only few studies on the effects of the combination of phytoestrogens with calcitriol have been published. Swami et al. [16] showed that genistein potentiates the action of calcitriol in human prostate cancer cells, and Rao et al. [17] demonstrated that these substances synergistically inhibit the growth of human prostatic epithelial cells. This was achieved by two related important mechanisms: 1) by directly inhibiting CYP24A1 enzyme activity, leading to an increase in the half-life of calcitriol (adults 5–8 h, children 27 h), and 2) by amplifying the homologous up-regulation of the vitamin D receptor (VDR) [18]. However, to our knowledge there are no reports on the effects of a combination of calcitriol and genistein on bone cancer cells. This is of interest, because a synergistic action of both substances is involved in the prevention of osteoporosis and the reduction of hip fracture risk in postmenopausal women [19]. Therefore, we hypothesize that genistein in the presence of calcitriol mediates synergistic anti-tumor activity in human bone cancer cells by distinct cell biological mechanisms. In the present study these hypotheses were elucidated by focusing on cell cycle analysis, metabolic alterations and signaling cascades.

## Material and Methods

### Chemicals

Genistein (4′,5,7-Trihydroxyisoflavone) and calcitriol (1α,25-Dihydroxycholecalciferol) were purchased from Sigma Aldrich (Germany) were stored at -20°C in the dark as single-used aliquots of concentrated stock solutions in dimethylsulfoxide (DMSO, for genistein) or ethanol (EtOH, for calcitriol).

### Cell culture conditions

The osteosarcoma cell lines MG-63 (ATCC^®^ CRL-1427™) and Saos-2 (ATCC^®^ HTB-85™) were obtained from the American Type Culture Collection (ATCC, Manassas, VA, USA). The human non-tumorigenic, primary osteoblast cells (POB) were chosen as control cells. Briefly, the primary osteoblast cells were isolated from the spongiosa of the femoral heads of patients undergoing primary total hip replacement. The samples were collected with patient agreement and approval by the Local Ethical Committee named “Ethikkommission an der Medizinischen Fakultät der Universität Rostock”, located at St.-Georg-Str. 108, 18055 Rostock, Germany with the registration number: A 2010–10 (see: https://ethik.med.uni-rostock.de/). The participants provide their written consent to participate in this study.

All cells were grown in Dulbecco’s modified Eagle’s medium (Invitrogen, Germany) with 10% fetal bovine serum (PAN Biotech GmbH, Germany) and 1% gentamycin (Ratiopharm, Germany). Application of genistein (final concentrations: 1, 10, 100 μM) and/or calcitriol (10 nM) was carried out in assay medium: phenol-red-free Dulbecco’s modified Eagle’s medium (PAA Laboratories GmbH, Germany) with 10% charcoal stripped fetal bovine serum (PAN Biotech GmbH, Germany) for 48 h. Prior treatment cells were adapted to the assay medium for 24 h.

### Cell cycle analysis

Cell preparation and cell cycle analysis have been described in detail previously (Engel et al., 2012, 2014). Cells in the cell cycle phases S and G2/M were summarized and defined as proliferative cells. Briefly, the extent of cell cycle progression and apoptosis in the cells was estimated by flow cytometric analysis after propidium iodide (Roche Diagnostics, IN, USA) staining. After treatment cells were trypsinized with 0.05% trypsin–0.02% EDTA for 5–10 min. The reaction was stopped with assay medium. Cells suspension was transferred to FACS tubes (BD Biosciences, USA) and fixed in 70% ethanol for 12 or more hours at −20°C. Briefly, after washing with PBS cells were incubated with RNase (1 mg/ml) at 37°C for 30 min. Finally, cells were re-suspended in propidium iodide (50 mg/ml) for at least 3 h at +2 to +8°C protected from light until flow-cytometric analysis. The software FlowJo version 10.0.5 (Tree Star Inc., USA) was used for data acquisition.

### Live cell metabolism

The live cell metabolic parameters cell impedance, oxygen consumption (respiration), and extracellular acidification were analyzed during stimulation with genistein and/or calcitriol for 24 h with the Bionas Discovery^TM^ 2500 system and the metabolic chip SC 1000 (Bionas GmbH, Rostock, Germany). Measurement conditions were already described [5]. Data sets were normalized to vehicle control treatment and set to 100% using the software Bionas1500^2^ Data analyzerV1.07.

### Immunofluorescence

The basic procedure for the immunofluorescence staining was described in Engel et al. (2012, 2014). Briefly, the rabbit anti-human estrogen receptor antibodies (ERα: sc-542, ERß: sc-8974), and the VDR (sc-13133, SGPL1: sc-67368), all from Santa Cruz, USA, were applied in a 1:50 dilution for 1 h at room temperature followed by the incubation of 488-labeled secondary goat anti-rabbit antibody (Molecular Probes, USA, 1:100) for 1 h at room temperature in the dark. Nuclei were counterstained with DAPI (Roche Diagnostics GmbH, Germany) for 15 min. Visualization and imaging was investigated with an inverted confocal laser scanning microscope (LSM780, Carl Zeiss, Germany) equipped with a helium/neon-ion laser and a ZEISS 63× oil immersion objective. Notably, photomicrographs stained with the same primary antibody were taken at identical exposure times and laser power to guarantee comparable results.

### Western blot

General western blotting procedure using previously described protocols is illustrated in Warsow et al. and Engel et al. [20,21]. The following primary antibodies were used: Hexokinase I (C35C4) Rabbit mAb: #2024; PFKP (D4B2) Rabbit mAb: #8164; LDHA (C4B5) Rabbit mAb: #3582; PDH Pyruvate Dehydrogenase (C54G1) Rabbit: mAb #3205; Fumarase (D9C5) Rabbit mAb: #4567; SDHA (D6J9M) XP® Rabbit mAb: #11998; p-Akt (S473): 9271S; Akt (pan) (C67E7): 4691S; P-p44/42 MAPK (T202/Y204) (D13.14.4E) XP (R): 4370L; p44/42 MAPK (Erk1/2): 9102 (all from cell signaling, USA); and SGPL1: sc-67368, ER alpha (MC-20): sc-542; ER beta (H-150): sc-8974; VDR (D-6): sc-13133; PCNA (PC10): sc-56; beta-Actin (C4): sc-47778; Vinculin (G-11): sc-55465, from Santa Cruz, USA. They were incubated with gentle shaking at 4°C overnight in a dilution of 1:1000. Secondary antibody (Anti-rabbit or anti-mouse IgG, HRP-linked Antibody #7074 or #7076, from cell signaling, USA) was incubated at room temperature for 1 h. To ensure equal amounts of loaded total soluble proteins on Mini-PROTEAN® TGX Stain-Free™ gels (Bio-Rad, Germany), the band intensities were visualized and calculated by stain-free technology via a ChemiDoc™ MP imager (Bio-Rad, Germany). Normalization and calculation were performed with Image Lab 3.0.1 (Bio-Rad, Germany). Protein signals were visualized by using SuperSignal West Femto Chemiluminescent Substrate (Pierce Biotechnology, Rockford, USA) for detection of peroxidase activity from HRP-conjugated antibodies (Thermo Fisher Scientific Inc., Rockford, USA). Band intensity was analyzed densitometrically with the Molecular Imager ChemiDoc XRS and Image Lab 3.0.1 software (Bio-Rad, USA). Protein detection was repeated at least three to five times with individually prepared cell lysates from independently passaged cells.

### Phosphorylation status of signaling proteins

Phosphorylated proteins (Phosph-ERK1/2 (T202/Y204, T185/Y187); No. 5016616, Phospho-Akt (S473); No. 5017459, Phospho-p38 MAPK (T189/Y182); No. 5017183, and Bio-Plex® Phosphoprotein Detection Reagent Kit; No. 310013270, all from Bio-Rad, Germany) were analyzed with the Bio-Plex 200 Array System with Bio-Plex™ Manager 4.1.1. software (Bio-Rad, Germany) according to the manufacturer's recommendations. Total protein lysates (10 μg soluble protein) were prepared with cell lysis kit from Bio-Rad, Germany. Protein lysates were incubated overnight in 96-well plates with fluorescent capturing beads. Then, plates were washed and incubated with biotinylated antibodies against the phosphorylated proteins and final added streptavidin-phycoerythrin solution. The measurement of phosphorylated proteins was performed with the relative mean fluorescence intensity calculated excluding the blank (approach without protein lysates) (n = 3).

### Metabolic profiling via GC-MS

Sample preparation was carried out according to the cell sampling protocol of mammalian adherent cells of Metabolomic Discoveries GmbH (Potsdam, Germany). Briefly, approximately 1 x 10^7^ cells adherent MG-63 cell were cultivated and treated with the vehicle control, genistein and/or calcitriol in T25 flasks (Greiner, Germany). After 48 h treatment flasks were put on ice, washed three times with ice-cold 0.9% (w/v) NaCl solution (saline) and thereafter cells were quenched with 500 μl ice-cold extraction buffer containing 80% methanol and removed from the flasks with the help of a cell scraper. Additional 500 μl extraction buffer were used to remove all cell remnants. Cell suspension was filled in cryo tubes and shock-frozen in liquid nitrogen. Samples were stored at -80°C until metabolic measurement. All subsequent steps were carried out at Metabolomic Discoveries GmbH (Potsdam, Germany; www.metabolomicdiscoveries.com)

300 μl of cell extract were used for global metabolite profiling analysis. Derivatization and analyses of metabolites by a GC-MS 7890A mass spectrometer (Agilent, Santa Clara, USA) were carried out as described [22]. Metabolites were identified in comparison to Metabolomic Discoveries' database entries of authentic standards. LC-MS analysis was performed using hydrophilic interaction chromatography with a ZIC-HILIC 3.5 μm, 200 A column (Merck Sequant, Umeå, Sweden), operated by an Agilent 1290 UPLC system (Agilent, Santa Clara, USA). The LC mobile phase was a linear gradient from 90% to 70% acetonitrile over 15 min, followed by linear gradient from 70% to 10% acetonitrile over 1 min, 3 min wash with 10% and 3 min reequilibration with 90% acetonitrile. The flow rate was 400 μl/min, injection volume 1 μl. Mass spectrometry was performed using a 6540 QTOF/MS detector (Agilent, Santa Clara, USA). The measured metabolite concentration was normalized to the internal standard C13-Sorbitol.

### Characterization of osteoblastic phenotypes

Alkaline phosphatase (ALP) specific activity was used as an early marker of the osteoblastic phenotype. Therefore, cells were washed in PBS and fixed in 4% paraformaldehyde for 5 min. After washing, cells were incubated with 0.1% naphthol AS-MX phosphate and 0.1% fast red violet LB salt in a 2-amino-2-methyl-1,3-propanediol buffer (56 mM) for 10 min. Alizarin red S staining to visualize calcification was performed according to manufacturer’s protocol (Osteogenesis assay kit, #ECM815, Millipore). Matrix mineralization was determined by immunofluorescence labeling with anti-osteocalcin (#AB10911, Millipore). As secondary antibody Alexa 488-labeled secondary goat anti-rabbit antibody (Molecular Probes, USA, 1:100) was used. After staining cells were counterstained with DAPI (Roche Diagnostics GmbH, Germany) for 15 min.

### Statistical analysis

All experiments were replicated at least three times with individually passaged cells, and data sets were expressed as means ± standard deviations (SD). Statistical significance was determined by the unpaired student’s *t*-test (*** P<0.001; ** P<0.01; * P<0.05). Significant concentration changes of metabolites in different samples were analyzed by appropriate statistical test procedures using the statistical analysis software R-project (www.r-project.org). Results of drug combination experiments (cell proliferation, receptor expression, metabolic alterations, SGPL1 expression) were analyzed for synergistic, additive, or antagonistic effects using primarily the coefficient of drug interaction (CDI) by the method of Chou-Talalay [23]. CDI calculations were done in custom Microsoft Excel templates as follows: CDI = AB/(A6B). According to the measurement values of each group, AB is the ratio of the combination groups to control group; A or B is the ratio of the single agent group to control group. Thus, CDI < 1, CDI = 1, and CDI > 1 indicate synergism, additive effects, and antagonism, respectively.

## Results

### Characterization of osteoblastic features

For analysis of putative synergistic effects of genistein and calcitriol on bone cancer cells, two tumorigenic osteosarcoma cell lines (MG-63, Saos-2) were compared to non-tumorigenic primary osteoblasts, isolated from patients’ cancellous bones (POB). MG-63 represents an early osteoblastic linage characterized by low alkaline phosphatase (ALP) activity and osteocalcin expression, while calcification, determined by Alizarin red staining, was comparable with Saos-2 cells (S1 Fig). In contrast, Saos-2 cells revealed the most mature osteoblastic profile: high ALP activity, calcification and mineralization rates. POBs exhibit the lowest osteoblastic differentiation levels identified by poor rates of ALP activity, calcification and mineralization.

### Calcitriol normalizes genistein induced proliferation promotion

**Fig 1:**
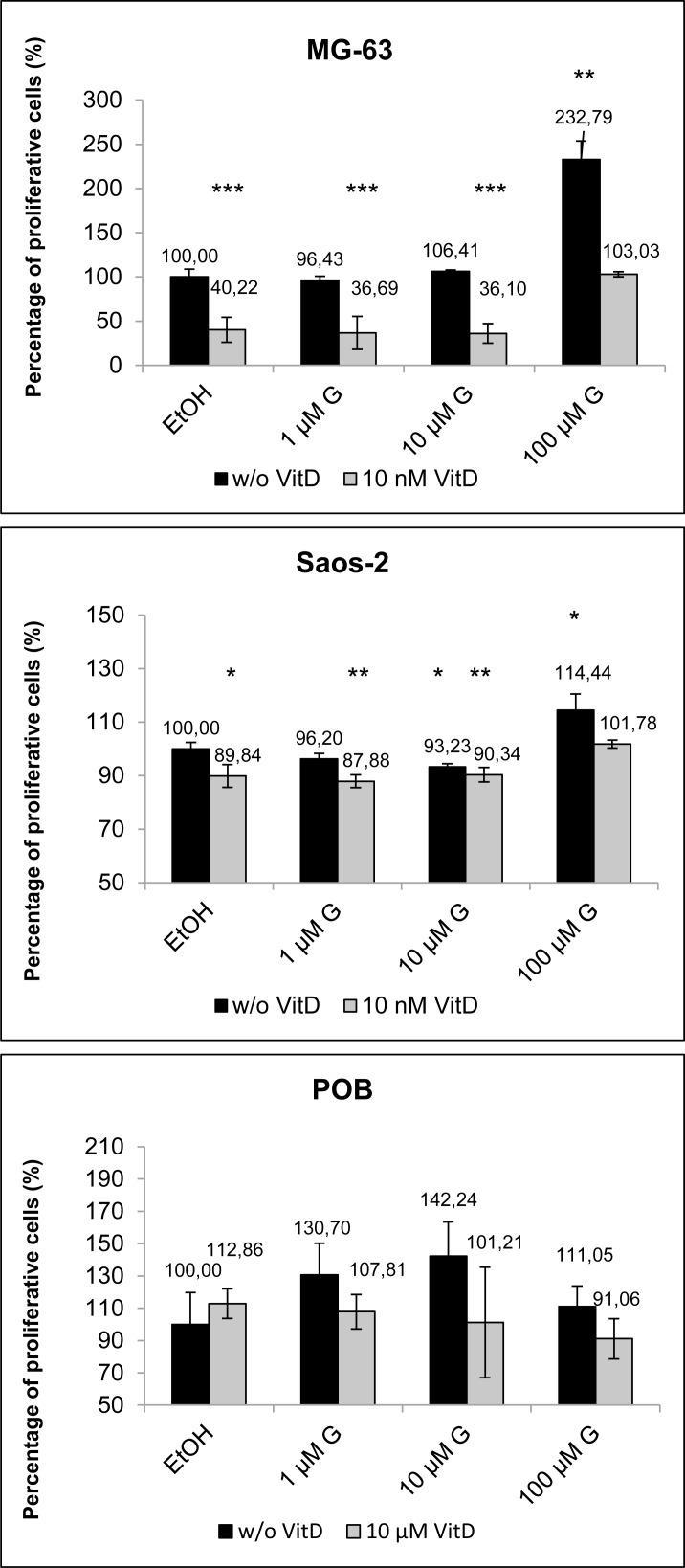
Cell cycle analysis of MG-63, Saos-2 bone cancer cells in comparison with primary osteoblasts (POB) after 48 h treatment with genistein (G; 1, 10, 100 μM; black bars) or in combination with 10 nM calcitriol (VitD; grey bars). Given is the percentage of proliferative cells (G2/M + S phase) whereby the control was set to 100%. Note that 100 μM genistein induces a strong proliferation induction in MG-63 cells which could be normalized to control levels by the addition of 10 nM calcitriol. Mean ± SD, n = 5, ***P < 0.001, **P < 0.01, *P < 0.5, significantly different compared to control, unpaired *t*-test.

Initially, the influence on cell proliferation after 48 h exposure to 10 nM calcitriol and a concentration series of genistein (1, 10, 100 μM), either separately or in combination, was tested via cell cycle analysis (Fig 1). Cells in the cell cycle phases G2/M and S were summed and marked as proliferative cells. The addition of 10 nM calcitriol reduced the proliferation of MG-63 (~ 60% reduction) and Saos-2 (~ 10% reduction) osteosarcoma cells. Treatment with 100 μM genistein only revealed significant proliferation induction in MG-63 (~ 130% induction) and Saos-2 (~ 14% induction) cells. After combined application of 100 μM genistein and 10 nM calcitriol proliferation was not different from untreated controls. This effect is particularly pronounced in MG-63 cells. Proliferation of POBs was not significantly altered after exposure to genistein, calcitriol or their combination, after treatment with genistein alone a trend towards slight proliferation induction was noted.

### Overexpression of ERß and VDR after combined treatment

**Fig 2:**
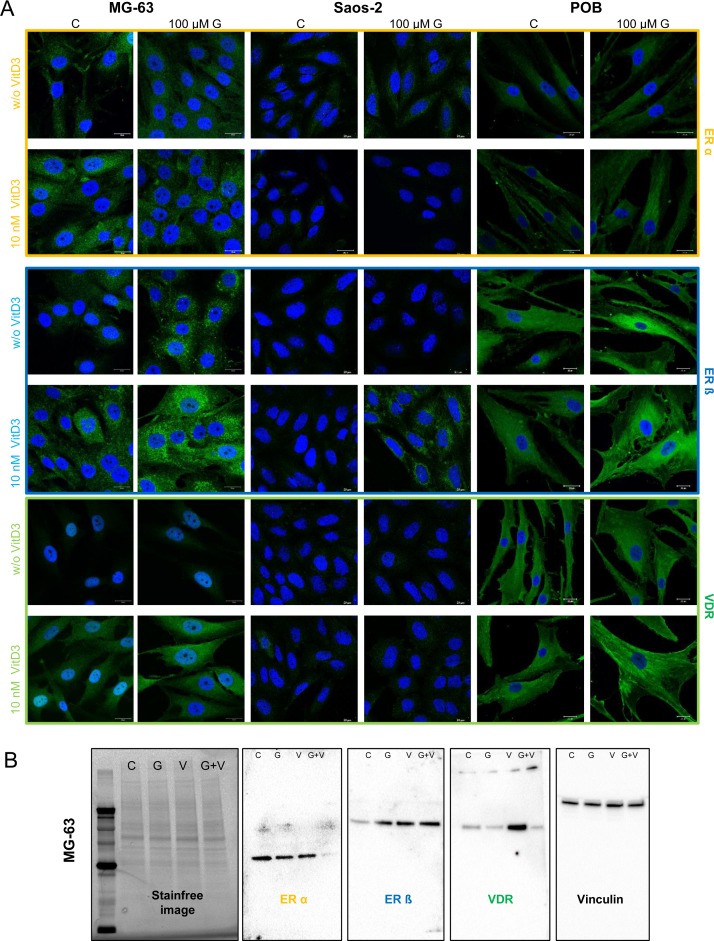
Immunofluorescence staining of ERα, ERß, and VDR in MG-63, Saos-2 and primary osteoblasts (POB) after 48 h exposure to the vehicle control (C), 100 μM genistein (G), 10 nM calcitriol (VitD3) or the simultaneous application of genistein and calcitriol. Receptor expression was secondarily labeled with Alexa488 (green). All samples were counterstained with DAPI to label the cell nucleus (blue). ERß and VDR expression is highly increased in MG-63 and POB cells after combined treatment with genistein and calcitriol. n = 3.

The cellular reactions to genistein are mediated by estrogen receptors (ERα, ERß), and calcitriol signaling is implemented by the VDR, functioning as hormone response elements on DNA resulting in expression or repression of specific gene products. Therefore, expression levels and cellular distribution of these three receptors were determined by immunofluorescence staining (Fig 2A) and Western blotting (Fig 2B). In MG-63, the combination of 100 μM genistein and 10 nM calcitriol caused significantly higher cytoplasmic expression levels of ERß as well as lower of ERα, visible in the immunofluorescence staining as well as in the western blot. While higher VDR expression in MG-63 was clearly visible by the increased fluorescence intensity, western blotting results only showed an increase of VDR after treatment with calcitrol. In the mature osteosarcoma cell line Saos-2 as well as in POBs only a slight increase of ERß expression was shown after simultaneous application. In summary, only the simultaneous application of genistein and calcitriol increased the ERß level in MG-63, primarily. This was the first hint, that combined treatment could reduce tumor progression in immature osteosarcoma cells.

### Synergistic influence on extracellular acidification and respiration

**Fig 3:**
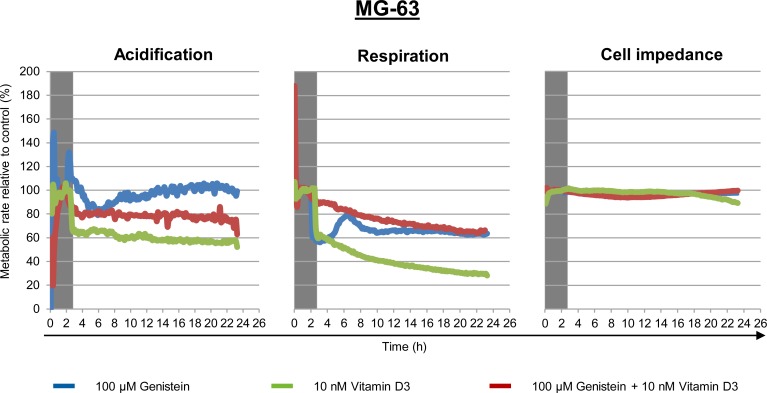
Live cell monitoring of three metabolic parameters (extracellular acidification, mitochondrial respiration, and cell impedance) in MG-63 after exposure to the vehicle control (was set to 100%), 100 μM genistein (blue lines), 10 nM calcitriol (red lines) or the combination of both (green lines) over 24 h. Prior treatment cells were adapted to the flow conditions for at least 2 h (grey shadowed). n = 3.

In order to elucidate this combinatory effects of genistein and calcitriol, metabolic real-time measurements were performed on MG-63 cells for 24 h (Fig 3). The Bionas Discovery^TM^ 2500 system and the metabolic chip SC 1000 enables an online monitoring of three cellular metabolic parameters: extracellular acidification (which is a measure of glycolytic flux in cancer cells), oxygen consumption (a measure of mitochondrial respiration), and cell impedance (a measure of cell adhesion, confluence and morphological alterations). Before treatment with genistein (100 μM), calcitriol (10 nM) or their combination, cells were adapted to the measuring conditions for 2–3 h until constant levels were achieved. The vehicle control values after adaption to the measuring medium were normalized and set to 100%. Extracellular acidification levels showed no alterations after exposure to genistein, a 20% reduction after treatment with calcitriol, and an additive reduction of 40% after combined treatment. A synergistic effect in respiratory levels could be observed after combinatory treatment with a reduction up to 80%. Cell impedance did not alter after any treatment. From this live cell monitoring could be concluded that MG-63 respiration and to a lower extent the glycolysis responded to the combinatory treatment of genistein and calcitriol. Therefore, expression studies of key enzymes of the glycolytic and respiratory pathways, important for cancer progression were conducted (S2 Fig). Protein expression levels of three glycolytic enzymes (hexokinase 1, PFKP: platelet-type phosphofructokinase, LDH: lactate dehydrogenase) and three enzymes of the Krebs cycle (PDH: pyruvate dehydrogenase, fumarase, SHDA: succinate dehydrogenase subunit A) after 48 h exposure to a concentration series of genistein only (1, 10, 100 μM; left panel) or a combined treatment with 10 nM calcitriol (right panel) in MG-63 cells were detected. The protein contents of these six metabolic enzymes changed only slightly (S2 Fig). But these slight alterations in enzyme content did not explain a reduction in respiration of 80% and in glycolysis of 40%. In order to identify the main trigger of the additive or synergistic action of genistein and calcitriol, metabolomic profiling in MG-63 cells was performed after 48 h exposure to the compounds.

### Synergistically elevated production of (phospho-) ethanolamine after combined treatment in MG-63 cells

On the basis of the proliferation results (Fig 1) and the live cell metabolic analysis (Fig 3) we decided to use the high concentrations of 100 μM genistein and 10 nM calcitriol for the following metabolomic profiling experiments. Four treatment groups were created: A: untreated control, B: treated with 100 μM genistein, C: treated with 10 nM calcitriol, D: treatment with both 100 μM genistein and 10 nM calcitriol. Subsequently to sample preparation, 186 metabolites were identified in this comprehensive analysis based on retention times, characteristic ions and mass spectra, comprising mainly amino acids, organic acids and lipids. To provide an initial overview of all analyzed samples, a principal component analysis (PCA) was calculated (S3 Fig). The PCA depicts 57.8% (46.6% + 11.2%) of all variances in this sample set. PC1 separates the controls (group A) and calcitriol-treated samples (group C) from the genistein (group B) and double-treated samples (group D). Thus, compared to the controls treatment with 100 μM genistein seems to have a larger influence on the metabolic profiles of the investigated samples than treatment with 10 nM calcitriol. However, potential additive or synergistic effects of individual metabolites caused by the combination of genistein and calcitriol cannot be deduced from the PCA.

**Fig 4:**
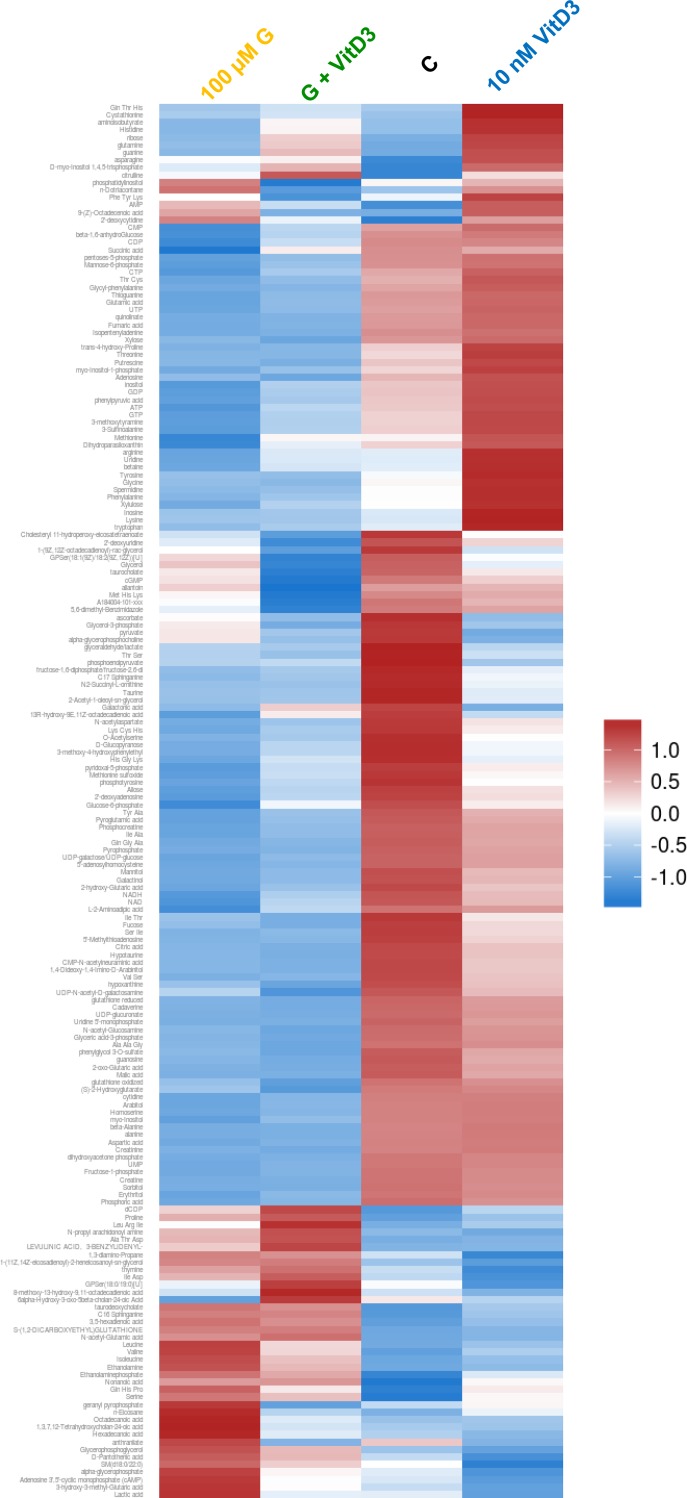
Heat map presentation of all identified metabolites by global metabolite profiling after 48 h treatment of the vehicle control, 100 μM genistein (G), 10 nM calcitriol (VitD3) and the combination of both in MG-63 cells (see color bar for scale; blue color indicates down regulated metabolites; red color indicates up regulated metabolites).

To give a more intuitive representation of differences for individual metabolite levels, a heat map based on hierarchical clustering for each metabolite was visualized (Fig 4). Up- and down-regulated metabolites are indicated by red and blue colors, respectively. It is clearly evident that compared to the control many metabolites after genistein or combined treatment are down-regulated (blue). But also a cluster of upregulated (red) metabolites in the left lower quadrant is visible. In the differential analysis, 103 metabolites showed a significant difference between 100 μM genistein and control treatment. Especially, components of various lipids are higher concentrated in the treated samples, whereas various amines and some amino acids show higher concentrations in the control. The comparison between 10 nM calcitriol and control treatment revealed 30 significantly altered metabolites, whereas amines, amino acids and lipids are higher concentrated in the calcitriol group. Compared to the control, 99 metabolites of the 100 μM genistein + 10 nM calcitriol group were significantly altered, this was comparable to the group treated with 100 μM genistein alone. In total, 186 compounds were analyzed with distinct changes in some metabolite classes based upon the treatment, e.g. lipid components, amines and amino acids. In energy metabolites, such as glucose or ATP, no significant changes were observed, strengthening the results of the protein expression analysis of relevant glycolytic and respiratory enzymes (S2 Fig).

**Fig 5:**
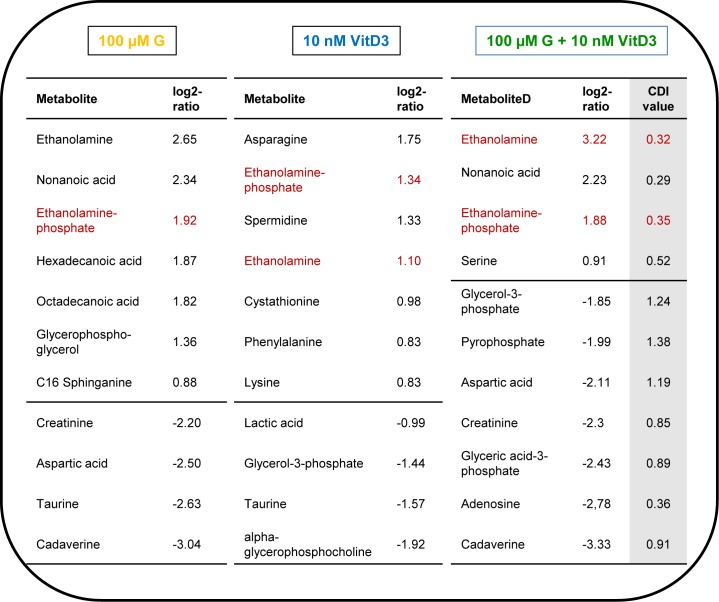
Ranking of metabolites which were significantly affected by genistein (G), calcitriol (VitD3) or the combination of both in comparison with control treatment (p ≤ 0.05; n = 4). Displayed were the log2 ratios. Notably, metabolites of the sphingolipid metabolism like ethanolamine and ethanolamine-phosphate were significantly upregulated. Synergistic effects of genistein and calcitriol were calculated by Chou-Talalay method, and displayed as CDI value. CDI < 1, CDI = 1, and CDI > 1 indicate synergism, additive effects, and antagonism, respectively.

To determine the significant altered metabolites, rankings of log2-ratios (treatment vs. control) were calculated (Fig 5). At first glance it is obvious that the single treatments with genistein and calcitriol regulate different metabolites. The application with genistein induces a strong increase in ethanolamine, nonanoic acid, ethanolamine-phosphate, hexadecanoic acid, octadecanoic acid and a boosted decrease in creatinine, aspartic acid, spermidine, taurine, and cadaverine. In contrast, calcitriol regulates the following metabolites: asparagine, ethanolamine-phosphate, spermidine and ethanolamine were up-regulated, and lactic acid, glycerol-3-phosphate, taurine, and α-glycerophosphocholine were down-regulated. The combined treatment revealed a strong increase for ethanolamine, nonanoic acid, and ethanolamine-phosphate whereas aspartic acid, creatinine, glyceric acid-3-phosphate, adenosine and cadavarine were significantly reduced. Synergism was calculated for ethanolamine, ethanolamine-phosphate and nonanoic acid.

Astonishingly, end products of the sphingolipid metabolism—ethanolamine and ethanolamine-phosphate—were upregulated synergistically after co-administration of genistein and calcitriol (Fig 5; highlighted in red). The production of ethanolamine-phosphate is irreversibly mediated by the sphingosine-1-phosphate lyase (SGPL1; UniProtKB—O95470; Gene ID: 8879), an enzyme cleaving the secondary messenger sphingosine-1-phosphate to (2E)-hexadecenal and ethanolamine-phosphate, which thereafter can be dephosphorylated by tissue-nonspecific alkaline phosphatases to ethanolamine. Due to this finding, expression and localization of SGPL1 were examined after stimulation with genistein and calcitriol.

### Upregulation of SGPL1 expression after combined treatment

**Fig 6:**
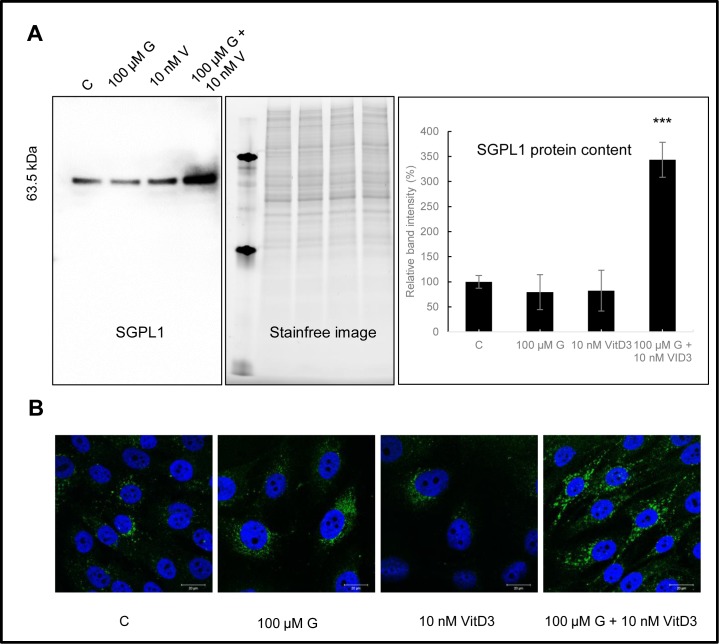
Protein expression analysis of the sphingosine-1-phosphate lyase (SGPL1) in MG-63 cells after treatment with the vehicle control (C), 1, 10, 100 μM genistein (G), 10 nM calcitriol (VitD3) or the combination of both via western blotting and immunofluorescence staining. **A:** western blot, stain free image as loading control and quantification of the western blotting results. Mean ± SD values (Representative example of 3 independent experiments, n = 3). *** = p ≤ 0.001 as compared to control (unpaired *t* test). B: Immunofluorescence staining of SGPL1 protein to compare expression levels und subcellular distribution. Green: SGPL1. Blue: cell nucleus. n = 3.

In MG-63 cells, SGPL1 expression studies after treatment with genistein, calcitriol or their combination were performed via western blot and immunofluorescence staining (Fig 6). Both methods revealed a strong up-regulation of SGPL1 protein content only after simultaneous stimulation with genistein and calcitriol, while SGPL1 expression levels were not changed after the single application of either substance. The western blot results indicate an increase of SGPL1 protein up to 350% compared to the control (100%) (p<0.001). Immunofluorescence staining of SGPL1 protein confirmed its subcellular localization in the endoplasmic reticulum membrane (http://compartments.jensenlab.org) as well as the overexpression after combined treatment (higher green fluorescence intensity (Fig 6)).

## Discussion

In this study for the first time the synergistic antitumor effects of genistein and calcitriol were demonstrated in cancer cells (osteosarcoma cells MG-63), which is associated with the expression up-regulation of sphingosine-1-phosphate lyase (SGPL1). The peculiarity of this finding is that the combination of both substances counteracts the pro-proliferative action of genistein in bone cancer cell lines. In detail, the application of phytoestrogens is discussed controversially because they can convey estrogenic as well as anti-estrogenic responses that thus may affect the growth and tumorigenicity of hormone-dependent tumors. High doses of genistein (>10 μM) can reduce the proliferation of breast cancer cells, but also at the same time they promote the growth of bone cancer cells [9]. We explored the co-administration of genistein and calcitriol because there is preliminary data that they could synergistically inhibit cancer growth [16, 17]. Indeed, the proliferation promotion induced by 100 μM genistein in the bone cancer cell line MG-63 could be normalized to control levels after simultaneous exposure to 10 nM calcitriol (Fig 1). Also, a significantly increased expression of the ERß could be observed while the expression levels of the ERα remained unchanged (Fig 2). Similarly, a significant increase of the VDR levels was determined in MG-63 cells and primary osteoblasts. This suggests that the combination of genistein and vitamin D3 stimulates an overexpression of ERß and VDR which are meant to be tumor suppressors and good prognostic markers [24, 25].

Beside the synergistic effects on the proliferation behavior and receptor expression, an influence on the cellular metabolism could be determined by live cell monitoring of three cancer relevant features: extracellular acidification, mitochondrial respiration and cell impedance (Fig 3). Again, synergistic effects could be observed. In MG-63 cells extracellular acidification and respiration were significantly reduced. This may indicate a downregulation of both the glycolytic activity and the enzymes of the Krebs cycle. This however could not be confirmed by protein expression analysis of cancer-relevant glycolytic and respiratory enzymes in MG-63 (S2 Fig). Distinct alterations of the protein contents of e.g. lactate dehydrogenase, pyruvate dehydrogenase or succinate dehydrogenase, key players in the energy metabolism of cancer cells, were not observed [26]. The lack of a direct impact on the energy metabolism of MG-63 cells by genistein and/or calcitriol was also confirmed by metabolite analysis (Figs 4 and 5). Distinct changes in some metabolite classes based upon the treatment, e.g. lipid components, amines and amino acids could be detected, but no significant changes were observed in energy metabolites such as glucose or ATP. However, live cell monitoring experiments revealed pronounced synergistic effects of genistein and calcitriol especially for MG-63 and POBs: respiratory and glycolytic activities in MG-63 cells are greatly reduced and in POB respiration they are upregulated strongly. The impact on Saos-2 was moderate, probably because Saos-2 represents a mature osteoblastic phenotype compared to MG-63 and POBs (S1 Fig). Thus, it could be clearly demonstrated that an effect on O_2_-consumption and extracellular acidification could be reached although no direct influence on relevant metabolic energy enzymes or metabolites could be determined.

Therefore, a complex metabolic profiling of MG-63 cells was performed (Fig 4). After co-administration of genistein and calcitriol, 99 metabolites, primarily lipids and amino acids, were altered. Comparisons of the log2-ratios of significantly (p ≤ 0.05) up-regulated metabolites for the three treatment groups revealed, that ethanolamine was synergistically up-regulated after combined exposure to genistein and calcitriol (Fig 5). Schmitt et al. [27] just recently showed that the antitumor agent phorbol-ester stimulates release of ethanolamine from the metastatic basal prostate cancer cell line PC3 suggesting that elevated ethanolamine production could be associated with antitumor activity.

Beside ethanolamine, also high levels of ethanolamine-phosphate were detected. Both metabolites are components of the glycerophospholipid metabolism (KEGG pathway map 00564) and sphingolipid metabolism (KEGG pathway map 00600) and are directly related to each other. Briefly, sphingosine-1-phosphate lyase (SGPL1; KEGG enzyme EC 4.1.2.27) irreversibly cleaves sphingosine-1-phoshate (S1P), a signaling sphingolipid acting as second messenger, into hexadecenal and ethanolamine-phosphate [28]. The latter is dephosphorylated by a phosphoethanolamine/phosphocholine phosphatase (KEGG enzyme EC 3.1.3.75) in the presence of H_2_O. Up-regulation of ethanolamine-phosphate and ethanolamine was confirmed by expression analysis of the SGPL1 (Fig 6). Combined treatment with genistein and calcitriol caused a 3.5-fold overexpression of SGPL1 indicating a high breakdown of S1P.

High intracellular S1P levels are associated with increased migration and invasion of cancer cells, and S1P is 5–10 times higher in ovarian cancer patients [29]. A reduction of intracellular S1P levels could be achieved by up-regulating the internal expression and enzyme activity of SGPL1. However, the role of SGPL1 in bone and other cancers has not been directly examined until now. Degagné et al. [30] demonstrated that mice with intestinal epithelium-specific SGPL1 deletion developed phenotypes with pathological evidence of inflammation, colon shortening, high cytokine levels, S1P accumulation, and development of colon tumors. Furthermore, a down-regulation of SGPL1 was detected in most tumors, and over-expression of SGPL1 enhanced sensitivity to cisplatin, carboplatin, daunorubicin and etoposide [31]. Thus, SGPL1 is a potential target for pharmacological manipulation for the treatment of malignant, autoimmune, inflammatory and other diseases [32]. For the first time, synergistic effects by exposure to high doses of genistein in combination with calcitriol were demonstrated in osteosarcoma cells, primarily by a 3 – 4fold up-regulation of the SGPL1 protein content. This up-regulation mediated reduction of proliferation, boosted ERß and VDR expression and altered tumorigenic metabolism. This discovery opens new avenues in the treatment of cancer patients and should be underpinned by *in vivo* studies.

## Conclusion

In summary, combined treatment of the immature osteosarcoma cell line MG-63 with genistein and calcitriol results in: (1) a growth normalization of genistein induced proliferation, (2) overexpression of ERß, (3) reduction of extracellular acidification and respiration rates, (4) synergistically increased ethanolamine production, primarily initiated by a 3 – 4fold overexpression of the sphingosine-1-phosphate lyase. Actually, the co-administration of genistein and calcitriol is investigated in a murine xenograft model.

## Supporting Information

S1 Fig Alkaline phosphate activity (ALP), calcification via Alizarin Red S staining and matrix mineralization with osteocalcin immunofluorescence staining in untreated MG-63, Saos-2 bone cancer cells and in primary osteoblasts (POB). All cells were counterstained with DAPI to visualize cell nuclei.
(TIF)

S2 Fig Protein expression analysis via western blotting of cancer relevant enzymes of the glycolysis and Krebs cycle in MG-63 cells after 48 h treatment with the vehicle control (C), 1, 10, 100 μM genistein (G), 10 nM calcitriol (VitD3) or the combination of both. Glycolytic enzymes were hexokinase 1, platelet-type phosphofructokinase (PFKP), lactate dehydrogenase (LDH). Pyruvate dehydrogenase (PDH), fumarase and succinate dehydrogenase subunit A (SDHA) were chosen as representative enzyme of the Krebs cycle. n = 3. Stain free images were added to verify that identical soluble protein concentrations were loaded on the polyacrylamid gels.
(TIF)

S3 Fig Principal component analysis (PCA) of control (A), cells treated with 100 μM genistein (B), 10 nM calcitriol (C) and 100 μM genistein+10 nM calcitriol (D) with four replicates each.
(TIF)
