# Supplementary figures and images for: Synergistic Action of Genistein and Calcitriol in Immature Osteosarcoma MG-63 Cells by SGPL1 Up-Regulation
Source: PLoS One. 2017 Jan 26;12(1):e0169742. doi: 10.1371/journal.pone.0169742 (PMC5268493; doi:10.1371/journal.pone.0169742)

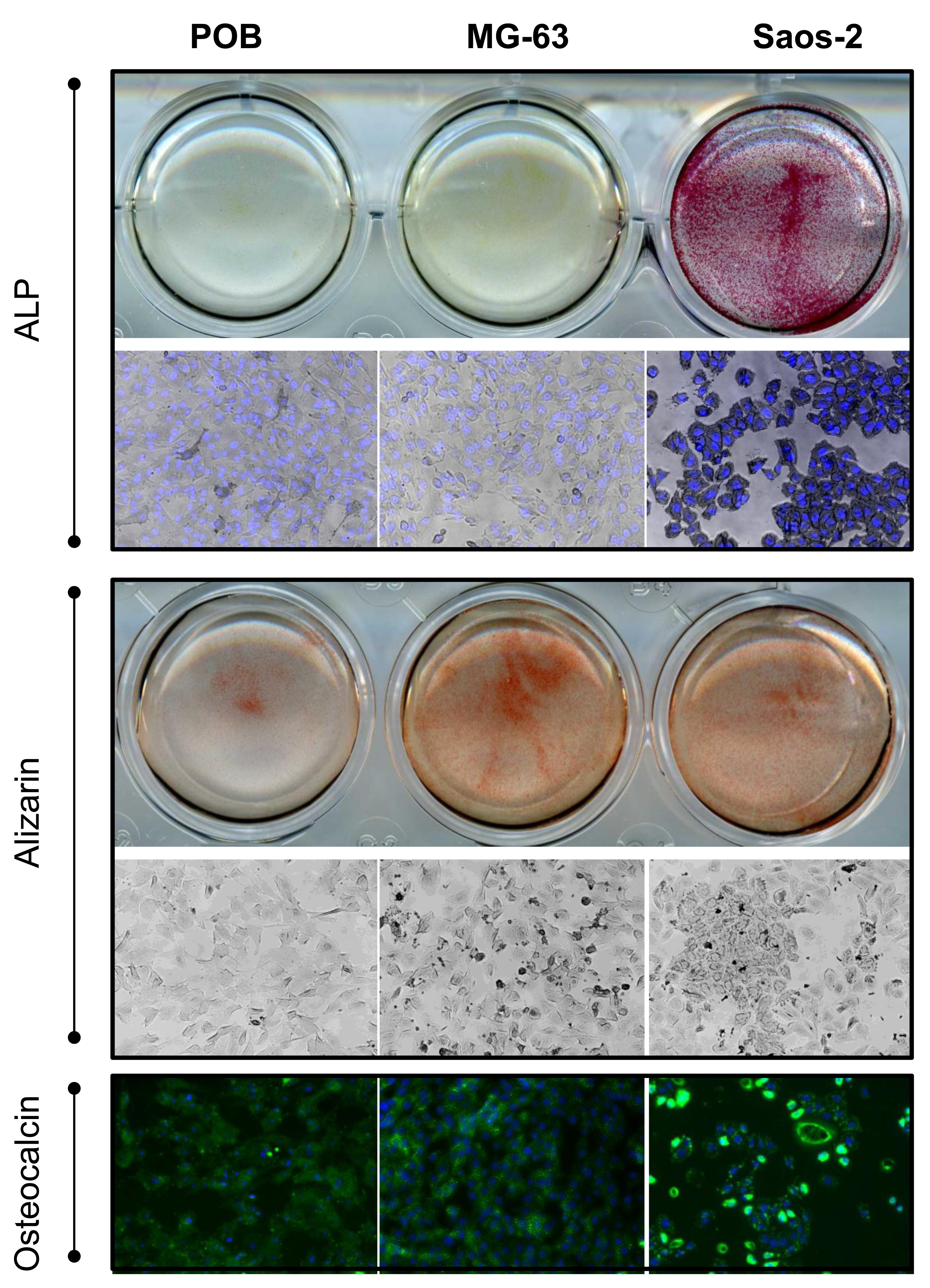

Supplement: S1 Fig — All cells were counterstained with DAPI to visualize cell nuclei. (TIF) [file pone.0169742.s001.tif]

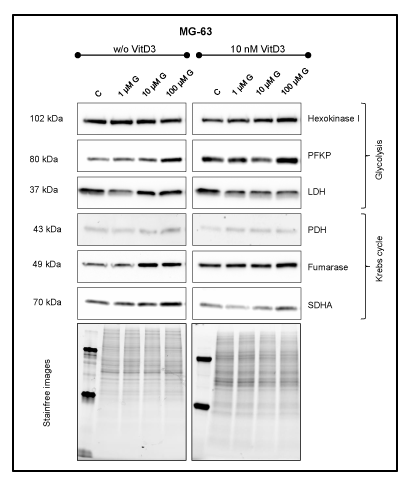

Supplement: S2 Fig — Glycolytic enzymes were hexokinase 1, platelet-type phosphofructokinase (PFKP), lactate dehydrogenase (LDH). Pyruvate dehydrogenase (PDH), fumarase and succinate dehydrogenase subunit A (SDHA) were chosen as representative enzyme of the Krebs cycle. n = 3. Stain free images were added to verify that identical soluble protein concentrations were loaded on the polyacrylamid gels. (TIF) [file pone.0169742.s002.tif]

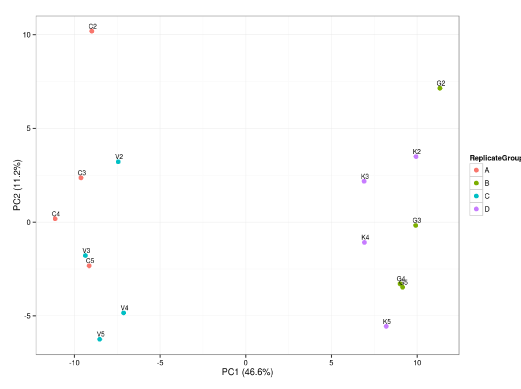

Supplement: S3 Fig — Principal component analysis (PCA) of control (A), cells treated with 100 μM genistein (B), 10 nM calcitriol (C) and 100 μM genistein+10 nM calcitriol (D) with four replicates each. (TIF) [file pone.0169742.s003.tif]
